# Evaluating the effect of a web-based quality improvement system with feedback and outreach visits on guideline concordance in the field of cardiac rehabilitation: rationale and study protocol

**DOI:** 10.1186/s13012-014-0131-y

**Published:** 2014-12-31

**Authors:** Mariëtte M van Engen-Verheul, Nicolette F de Keizer, Sabine N van der Veer, Hareld MC Kemps, Wilma JM Scholte op Reimer, Monique WM Jaspers, Niels Peek

**Affiliations:** Department of Medical Informatics, Academic Medical Centre/University of Amsterdam, (Room J1b-113.2), P.O. Box 22660, 1100 DD Amsterdam, The Netherlands; European Renal Best Practice (ERBP) Methods Support Team, University Hospital Ghent, Ghent, Belgium; Department of Cardiology, Máxima Medical Centre, Veldhoven, The Netherlands; Amsterdam School of Health Professions, Amsterdam, The Netherlands; Health e-Research Centre, Institute of Population Health, University of Manchester, Manchester, UK

**Keywords:** Quality improvement, Quality indicators, Health care, Cardiac rehabilitation, Guideline adherence

## Abstract

**Background:**

Implementation of clinical practice guidelines into daily care is hampered by a variety of barriers related to professional knowledge and collaboration in teams and organizations. To improve guideline concordance by changing the clinical decision-making behavior of professionals, computerized decision support (CDS) has been shown to be one of the most effective instruments. However, to address barriers at the organizational level, additional interventions are needed. Continuous monitoring and systematic improvement of quality are increasingly used to achieve change at this level in complex health care systems. The study aims to assess the effectiveness of a web-based quality improvement (QI) system with indicator-based performance feedback and educational outreach visits to overcome organizational barriers for guideline concordance in multidisciplinary teams in the field of cardiac rehabilitation (CR).

**Methods:**

A multicenter cluster-randomized trial with a balanced incomplete block design will be conducted in 18 Dutch CR clinics using an electronic patient record with CDS at the point of care. The intervention consists of (i) periodic performance feedback on quality indicators for CR and (ii) educational outreach visits to support local multidisciplinary QI teams focussing on systematically improving the care they provide. The intervention is supported by a web-based system which provides an overview of the feedback and facilitates development and monitoring of local QI plans. The primary outcome will be concordance to national CR guidelines with respect to the CR needs assessment and therapy indication procedure. Secondary outcomes are changes in performance of CR clinics as measured by structure, process and outcome indicators, and changes in practice variation on these indicators. We will also conduct a qualitative process evaluation (concept-mapping methodology) to assess experiences from participating CR clinics and to gain insight into factors which influence the implementation of the intervention.

**Discussion:**

To our knowledge, this will be the first study to evaluate the effect of providing performance feedback with a web-based system that incorporates underlying QI concepts. The results may contribute to improving CR in the Netherlands, increasing knowledge on facilitators of guideline implementation in multidisciplinary health care teams and identifying success factors of multifaceted feedback interventions.

**Trial registration:**

NTR3251.

## Background

Concordance to clinical practice guidelines can improve patient outcomes, reduce practice variation, and reduce costs of health care [1-3]. However, implementation of guidelines into daily care is hindered by a variety of barriers related to decision-making behavior of health care professionals and collaboration in teams and organizations [4]. An electronic patient record (EPR) information system with computerized decision support (CDS) functionalities was previously developed to stimulate guideline implementation in cardiac rehabilitation (CR) in the Netherlands [5,6]. Although the CDS system was effective in improving concordance with the guidelines recommendations, there remained a considerable non-concordance due to organizational constraints (e.g., lack of time or resources) [7]. Therefore, we hypothesize that guideline implementation with CDS systems directed at professionals may be more powerful if used in conjunction with other interventions directed at the decision-making processes at the organizational level [8]. This paper describes the design of an evaluation study of a multifaceted intervention combining indicator-based performance feedback and educational outreach visits to improve guideline implementation in a context of multidisciplinary CR teams working with an EPR with CDS already.

### The problem: persisting external barriers to guideline implementation

Poor concordance to clinical practice guidelines is due to various barriers that professionals may face when they try to incorporate practice guidelines into daily care. An often-used classification of those barriers is the division into internal and external barriers [9]. Internal barriers relate to professional knowledge of, and attitudes towards, the guidelines. For instance, professionals may not be familiar with the details of a guideline or may disagree with its recommendations. However, because health care professionals often work within complex organizations, appropriate knowledge and attitudes of professionals are necessary but not sufficient to implement guidelines. Professionals may also encounter external barriers which hamper their ability to execute guideline recommendations. These barriers concern environmental factors related to the organization or health system professional work in, such as a lack of resources. External barriers also include barriers related to patients (e.g., patients may refuse therapies) and to the guidelines themselves (e.g., ambiguities, omissions, and contradictions).

Of the different implementation strategies to overcome barriers to guideline implementation (e.g., education, outreach visits, CDS, and reminders [10]), those providing patient-specific recommendations at the time and place where professionals make clinical decisions are most likely to be effective [2]. A recent systematic review and meta-analysis demonstrated that, in general, CDS increases the chance that recommended therapies are actually prescribed by 57% (odds ratio 1.57; 95% confidence interval 1.35 to 1.82) [11]. In individual trials, however, CDS is not always effective. A systematic review in the area of chronic disease management (including CR) shows that CDS led to significant improvements in the process of care in only 25 out 48 trials (52%) [12]. Attempts to identify critical success factors for CDS systems have provided inconsistent results, and it seems likely that these factors are highly dependent on the time and context of the intervention [13]. Overall, this seems to suggest that CDS is an effective instrument to overcoming internal barriers (i.e., related to clinical decision-making of professionals) to guideline implementation. However, external barriers are probably not addressed by CDS interventions.

This phenomenon that barriers faced by front-line professionals do not reach their managers and policy makers was also described by Tucker and Edmondson [14]. Clinicians may not be familiar with underlying concepts to address external barriers [15] and often lack time to be actively involved in activities other than patient care. This makes successfully overcoming external barriers a challenging endeavor. Therefore, we hypothesize that besides CDS, additional interventions are needed to also address external barriers related to decision-making processes at the organizational level of the health care clinic professional work in.

### Solving the problem: overcoming external barriers by providing a quality improvement intervention with performance feedback and outreach visits

**Figure 1:**
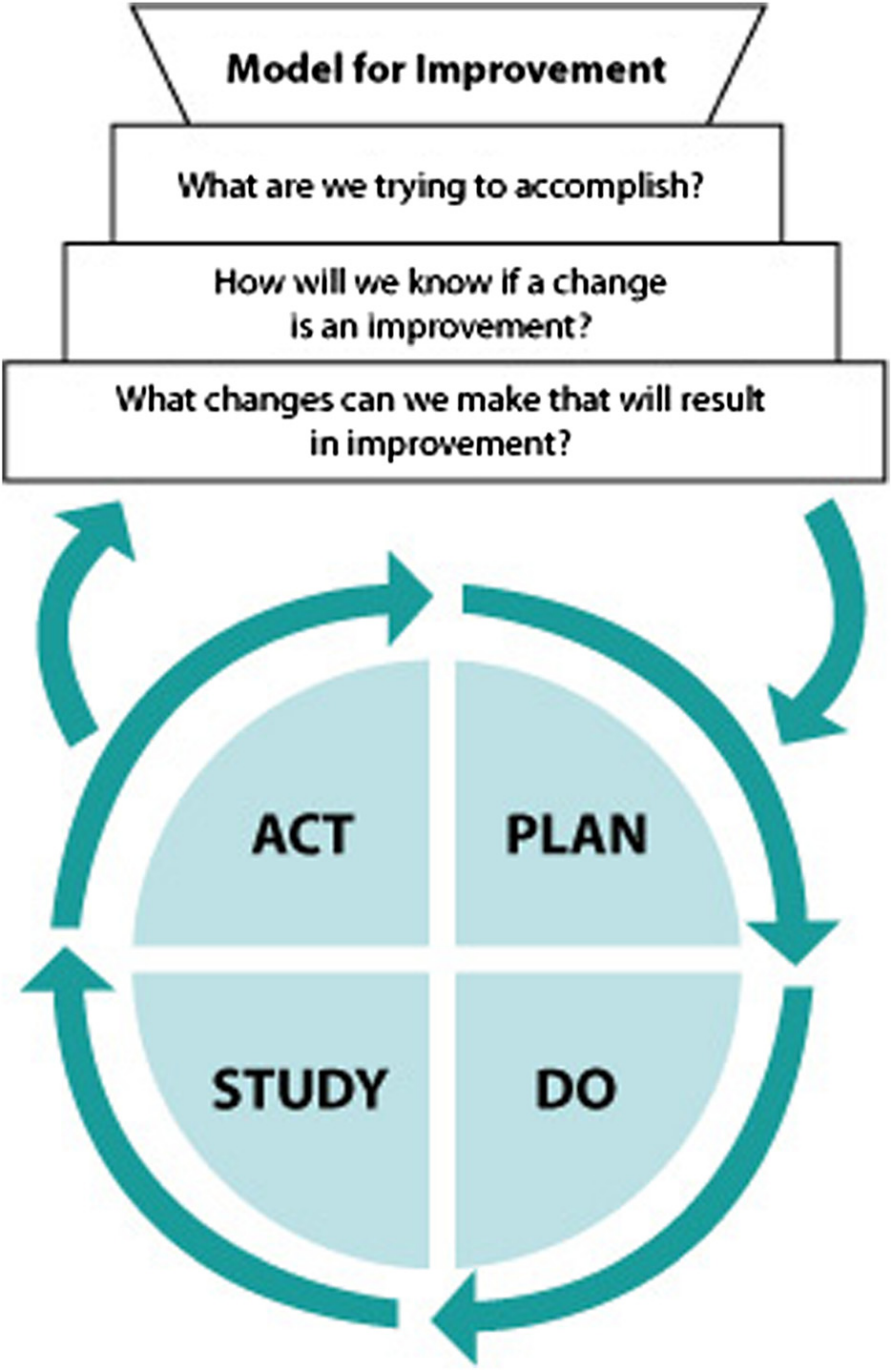
**The model for improvement [** 16 **].**

A common approach to changing complex organizations is systematic quality improvement (QI), which focuses on improving an organization’s underlying processes. It relies on data from health care professionals’ own setting and encourages working in multidisciplinary QI teams. The teams’ performance should guide them in improving their practice by the plan-do-study-act (PDSA) cycle, a part of the Model for Improvement [16] (see Figure 1). Performance feedback is a crucial element within the plan and the study step in the PDSA cycle. Quality indicators—i.e., quantitative measures to monitor and evaluate the quality of health care processes that affect patient outcomes [17]—commonly serve as a basis for the feedback. The assumption is that it prompts professionals to change their behavior if they see that their practice does not meet benchmark values (e.g., national target values or average performance within a peer group). A recent Cochrane review on the effect of audit and feedback reported a median absolute increase in compliance with desired practice of +4.3% (interquartile range (IQR) 0.5% to 16%) on dichotomous measures (e.g., proportion of patients adhering to their therapy plan) and +1.3% (IQR 1.3% to 28.9%) on continuous measures (e.g., time between referral and intake). The reviewers suggested audit and feedback to be most effective if provided by a supervisor or colleague, more than once, both verbally and in writing, if baseline performance is low, and if it includes explicit targets and an action plan. Furthermore, the effect of indicator-based performance feedback is likely to be stronger when it is combined with educational meetings, directed towards actively involving care professionals in the improvement process [17].

Based on the literature on feedback, we hypothesize that complementing CDS with a performance feedback and outreach visit intervention might be an effective way to involve front-line professionals and their managers together in overcoming external barriers to improve their practice. This matches the conclusion of a substantial proportion (although not all) of systematic reviews concerning the effectiveness of different guideline-implementation interventions [10,18-21]. They indicate that effective strategies often have multiple components and that the use of single-component strategies is less effective [21]. Change is possible, but generally requires comprehensive approaches at different levels (doctor, team practice, hospital, wider environment), tailored to specific settings and target groups [20].

### Aim and objective

Given the positive effect of CDS on decision-making behavior of health care professionals but the remaining practice variation caused by organizational barriers, the development and assessment of an intervention tailored to external barriers to change are necessary. This study aims to intervene on factors influencing the implementation of guidelines at the organizational level in a setting where an existing CDS is used to guide professional decisions. We hypothesize that a web-based QI system with indicator-based performance feedback and educational outreach visits to multidisciplinary care teams will overcome the organizational constraints for changes needed to improve guideline concordance [22]. Table 1 describes the motivation of the elements included in the developed multifaceted intervention based on both the literature and from earlier studies performed in the field of CR by our research group.

**Table 1:** **Previous studies—improving guideline concordance in the field of CR**

**Study**	**Description of the studies**
I. *Tackling internal barriers: CDS*	To stimulate the implementation of the Dutch CR Guidelines, an EPR with CDS functionalities named CARDSS (cardiac rehabilitation decision support system) was previously developed [5]. After entering patient data, CARDSS provides its users with a patient-specific, guideline-based rehabilitation program, consisting of recommended rehabilitation goals and therapies. The effect of the system was evaluated in a cluster-randomized trial in 21 CR clinics, which showed that the system increased concordance to the CR guideline: CR professionals using the system better adapted the CR therapy to patients’ needs [7]. Data from the same trial however pointed out that there remained to exist a large variation in CR practice across clinics. For instance, the percentage of patients participating in exercise training varied from 41% in one clinic to 100% in another and the percentage of patients participating in education and counseling programs varied from 39% to 96%.
II. *Persisting barriers after introduction of CDS*	After the trial a qualitative study was conducted to investigate which barriers were reduced and which barriers persisted after introduction of the CDS system [8]. Results from semi-structured interviews with 29 CR professionals showed that the system succeeded in overcoming professional knowledge barriers. For instance, professionals were more aware of the need to use objective instruments to assess patients’ needs and of the therapy decision rules as described in the guidelines. However, two remaining barriers for guideline concordance frequently mentioned were organizational and guideline-related barriers; both can be classified as external barriers according to Cabana et al. [9].
III. *Tackling external guideline-related barriers: revision of the guidelines*	To overcome guideline-related barriers, the clinical algorithm for assessing patient needs in CR was revised [23]. We combined patient data collected by CARDSS and input from academic and practical experts. Assessment of patient needs based on clinical judgment was found to be a source of practice variation and was therefore avoided in the revised algorithm by adding several standardized assessment instruments.
IV. *Tackling external organization-related barriers: pilot study with feedback*	To address the remaining organizational-related barriers, a once-only benchmark-feedback loop was introduced in a pilot study in 21 clinics [22]. Data from the CDS system at different clinics were collected, stored in a central data registry, and used to generate paper feedback reports with benchmark information for each of the clinics. The reports aimed to steer discussions in team meetings, encouraging them to formulate QI plans. Although the reports were positively received by the clinics, many were unable to create time to discuss and actually act upon the report.
V. *Developing quality indicators*	For providing quality feedback to CR clinics, we developed a national preliminary set of quality indicators. This was performed in close collaboration with an expert (representatives from all disciplines involved in CR) and patient panel using a modified Rand method [24]. Within this method, results from both panels were combined with results from a literature search and guideline review in an extensive rating and consensus procedure. Table 3 shows the final set including 18 quality indicators regarding guideline concordance (e.g., complete data collection during needs assessment) and other quality aspects perceived relevant by both panels (e.g., patients participate in satisfaction research). Based on user experiences during this trial, we aim to select a subset of quality indicators to be rolled out on a national level.

**Table 3:** **Quality indicator set for cardiac rehabilitation per study arm**

**Study arm**	**Nr**	**Type**	**Quality indicator**
A	1	Structure	Specialized education for patients with chronic heart failure
	2a	Process	Complete data collection during needs assessment for rehabilitation (concerning psychological and social functioning, and lifestyle factors)
(Patient education, quality of life, and lifestyle change therapy [ex. physical activity])			
	3	Process	Patients receive a discharge letter to stimulate continuation of lifestyle changes at home
	4	Outcome	Patients quit smoking
	5	Outcome	Patients improved their quality of life during rehabilitation
B	2b	Process	Complete data collection during needs assessment for rehabilitation (concerning physical functioning and, cardiovascular risk factors)
(Exercise training and physical activity, relaxation and stress management, cardiovascular risk factors, and work resumption)			
	6	Process	Cardiovascular risk factors are evaluated after rehabilitation
	7	Outcome	Patients improve their exercise capacity during rehabilitation
	8	Outcome	Patients meet the physical activity norms
	9	Outcome	Amount of time needed to start resumption of work
A and B	10	Structure	Rehab professionals work with a multidisciplinary patient record
	11	Structure	Long-term patient outcomes are assessed
	12	Structure	Patients participate in patient satisfaction research
	13	Structure	Clinics perform internal evaluations and quality improvement
	14	Process	Average time between hospital discharge and start of rehabilitation
	15	Process	Patients are offered a rehabilitation program tailored to their needs
	16	Process	Patients finish their rehabilitation program
	17	Process	Rehabilitation goals are evaluated afterwards
	18	Process	Cardiologists receive a report after the rehabilitation

The key objective of the study is to assess the effectiveness of a web-based QI system with periodic performance feedback on quality indicators for CR and educational outreach visits to multidisciplinary QI teams to overcome organizational barriers for guideline concordance in the field of CR in the Netherlands. Primary outcome will be the impact of our intervention on concordance to the national CR guidelines (concerning CR needs assessment and therapy indication procedure). Secondary outcomes are changes in performance of CR clinics measured by changes in structure, process, and outcome indicators for CR and changes in practice variation on these indicators. A qualitative process evaluation (concept-mapping methodology) will be used to assess experiences from participating CR clinics and to gain insight into factors which influence implementation of the intervention. The results of this study can be used by those involved in QI of the CR care in the Netherlands. More in general, our study results may contribute to a better understanding of factors that influence the implementation of practice guidelines and can be used to set up multifaceted guideline implementation programs in other fields of health care.

## Methods

### Study design

**Figure 2:**
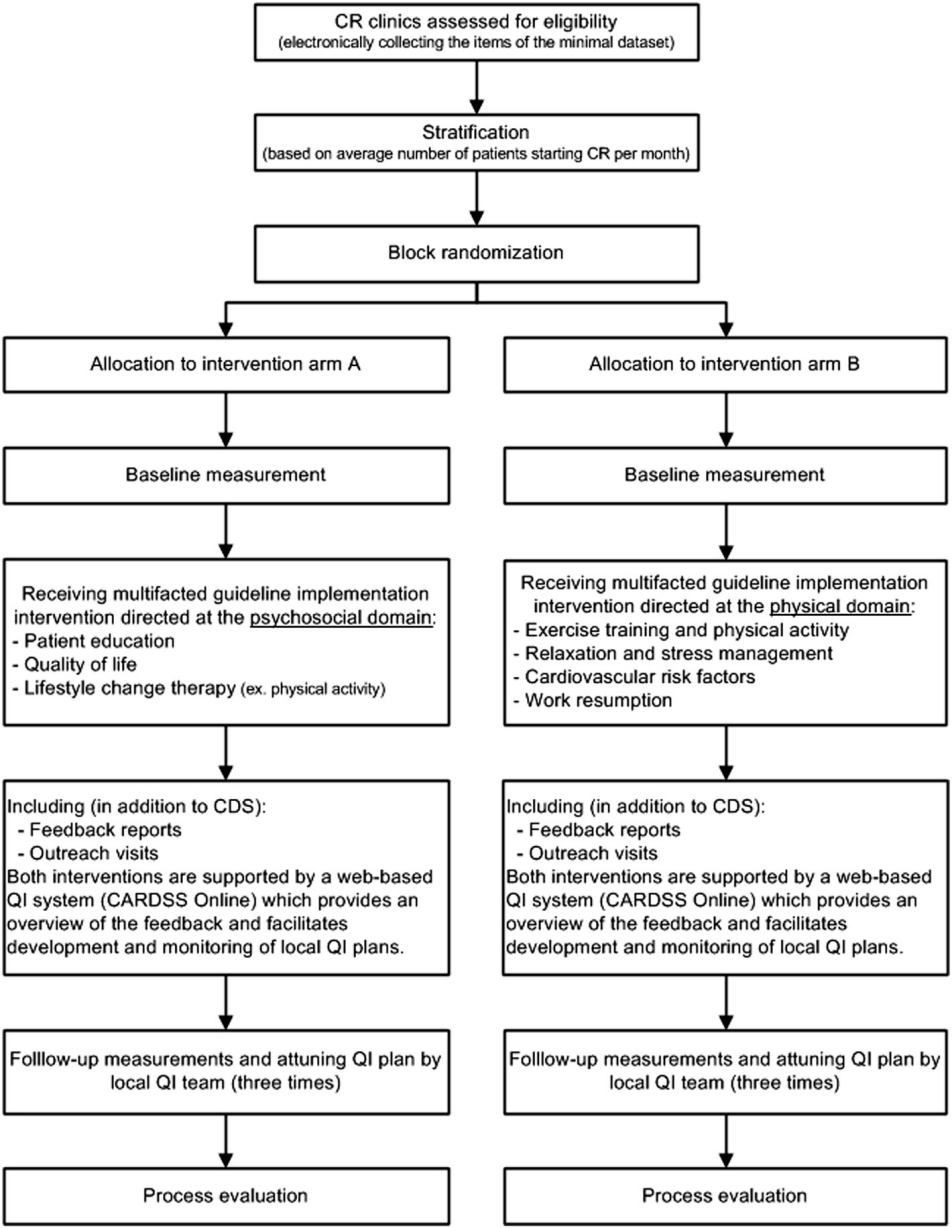
**Study flow.**

The effect of the intervention will be evaluated in a multicenter cluster-randomized study with a balanced (2 × 2) incomplete block design. Cluster-randomization is chosen to avoid contamination among professionals within the same clinic [25]. During the trial, clinics will be divided into two study arms (A and B). Using the multidisciplinary character of CR treatment, each arm will receive the intervention (a web-based QI system with feedback and educational outreach visits) directed at one out of the two domains described in the CR guidelines [26]. Clinics allocated in arm A will receive the intervention directed at improving guideline concordance for the psychosocial domain, clinics in arm B for the physical domain (see Figure 2 for an overview of the study flow). In this way, both study arms will serve as each other’s control. For all participating clinics, the study period is 1 year.

### Clinical setting: cardiac rehabilitation in the Netherlands

The study will take place in Dutch CR clinics that already work with an EPR with CDS at the point of care. CR is a therapy provided by multidisciplinary care teams to support heart patients recover from a cardiac incident or intervention, and aims to improve their physical and psychological condition [27-31]. CR is recommended for all patients who have been hospitalized for an acute coronary syndrome (ACS) and for those who have undergone coronary revascularization (coronary artery bypass graft surgery [CABG] or percutaneous coronary interventions [PCI]) or valvular surgery [32,33]. Recent studies show that CR is also beneficial for patients with other chronic cardiovascular conditions such as stable angina pectoris (AP) and chronic heart failure (CHF) and for subjects with a high risk for developing cardiovascular disease [34]. A recent meta-analysis shows consistent evidence of the effectiveness of exercise-based and multimodal (e.g., psychosocial and stress management) CR interventions with regard to mortality and prevention of future cardiac events (relative-risk reduction 21–47%) [35]. CR teams usually include cardiologists, physical therapists, nurses, psychologists, dieticians, social workers, and rehabilitation physicians. It has proven to be cost-effective in different economic evaluations conducted in North America and Europe [29]. However, in many Western countries, CR services are under-utilized and poorly standardized and do not follow the available scientific evidence [36]. A recent study in the Netherlands shows that only a minority of patients eligible for CR actually receive it [37]. The CR uptake rate was 28.5% among patients with an ACS and/or intervention. From these, patients who underwent CABG or valve surgery had the highest uptake rate (58.7%) and patients with an ACS without an intervention had the lowest uptake rate (9.8%) [37].

### Context: the use of CDS in participating CR clinics

All participating CR clinics use an EPR with CDS based on the most recent version of the CR guidelines [26]. The CDS provides advice on a patient-tailored rehabilitation program based on the needs assessment procedure. This procedure requires gathering 80 to 130 data items concerning the patient’s health status and rehabilitation needs. A clinical algorithm describing a branching logic to assess the data is part of the guidelines [38]. The rehabilitation program can contain four possible group-based therapies: exercise training (optimize exercise capacity with physical restrictions); education therapy (about consequences of the patient’s disease); lifestyle change therapy (risk-related behavioral adjustment); relaxation and stress management training (learning to manage tension in daily practice); and if needed, different forms of individual therapy (e.g., by psychologists). During the needs assessment procedure, the CDS advice can immediately be discussed with the patient to set the final rehabilitation program.

### Intervention

**Figure 3:**
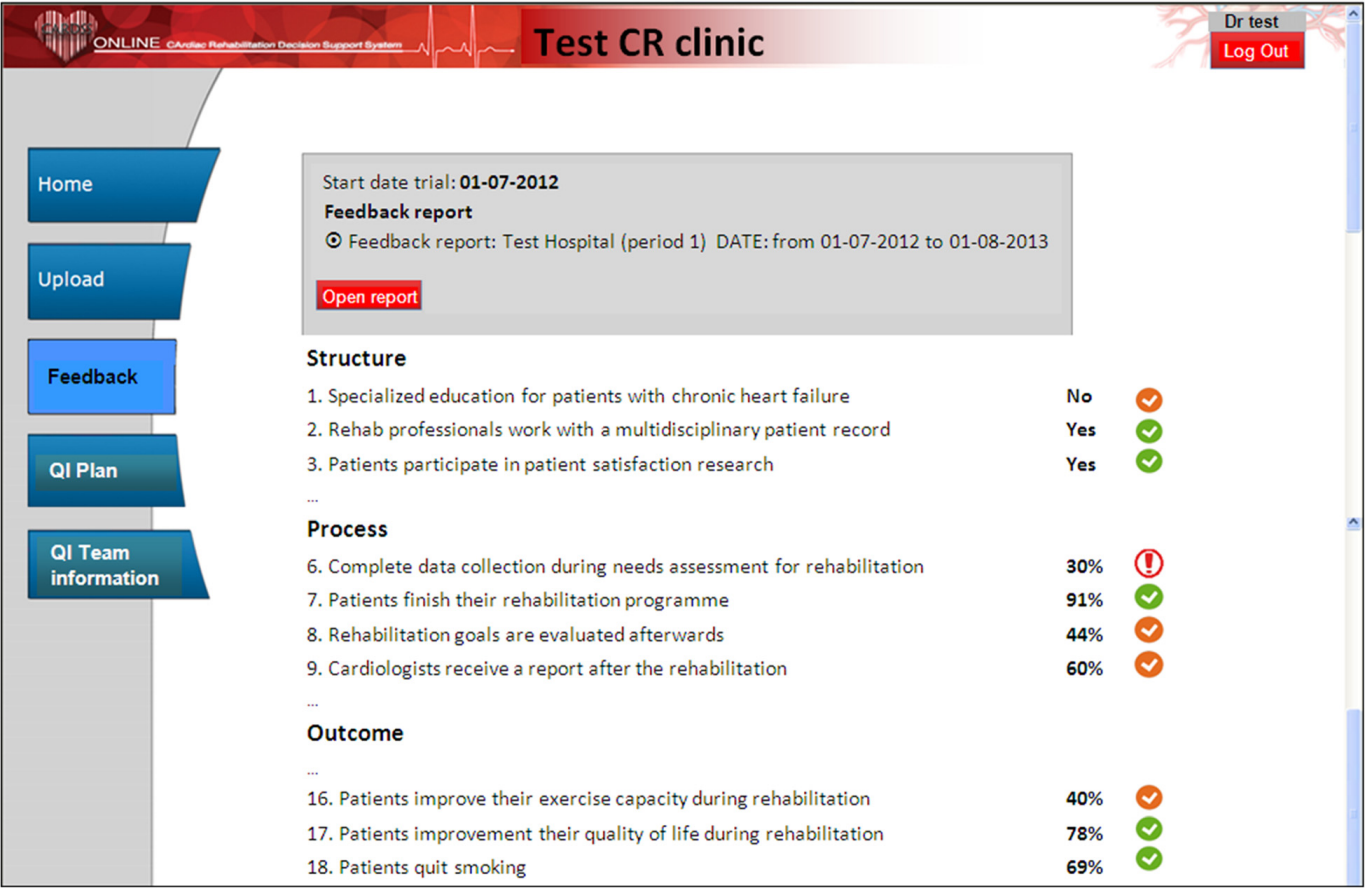
**Screenshot CARDSS Online (feedback report).**

We will carry out a multifaceted guideline implementation intervention that consists of two elements: (i) quarterly feedback on quality indicators for CR, provided as part of a web-based QI system and (ii) educational outreach visits using the same system to set up a QI plan together with a local QI team. Table 2 gives an overview of the complete intervention for both study arms.

i.) Feedback. Each 3 months, all participating clinics will receive feedback on a set of quality indicators for CR [24], based on data that are recorded by the CDS system. Feedback reports will be presented within the web-based QI system called CARDSS Online, which was specifically developed for this study [39]. Within this system, clinics can monitor their performance on the quality indicators listed in Table 3. Figure 3 shows a screenshot from the feedback report page in CARDSS Online. After clicking on an indicator button, a pop-up screen opens with detailed information about data underlying the calculation, national averages for comparison, and benchmark values. Feedback will be attuned to the research arm concerned: clinics allocated in arm A will receive feedback on quality indicators referring to psychosocial domain; clinics allocated in arm B will receive feedback on indicators referring to the physical domain. Both arms will receive feedback on indicators referring to general processes and structures (see Table 3).
  To assist the QI teams with selecting the quality indicators requiring improvement, a colored icon next to each indicator score indicates whether the performance is acceptable (green checkmark), borderline (orange checkmark), or poor (red exclamation mark). In addition, results will be compared to statistics from the same clinic during earlier periods to quantify change. When a clinic’s average for a given indicator is poor or differs (both in positive and negative sense) from previous results, this will be discussed during the outreach visits. Icon colors are automatically determined by the CARDSS Online system, using predefined rules (see Appendix I).
ii.) Outreach visits*.* Feedback reports will be received by all participating clinics on a 3-month basis and followed by outreach visits to support interpretation of the report and to draft a QI plan. This approach was chosen to match the conclusion from the Cochrane review that the feedback may be more effective when it includes both an action plan and explicit goals [17]. During development of the QI plan content, additionally, the goal-setting theory, stating that feedback and well-specified goals are indeed a successful combination, was used. The theory emphasizes that people tend to be more committed to attaining a certain goal if they are involved in setting it, if (the outcome of) goal attainment is seen as important, and if people believe they are capable of accomplishing it [40]. To this end, each clinic needs to set up a local QI team with the responsibility to define, implement, and monitor a QI plan including concrete QI goals based on self-identified issues in the feedback reports.
  QI team: Setting up a QI team implies the allocation of at least two CR team members for an average of 3 h per month (anyhow, the nurse acting as rehabilitation coordinator and one person from another discipline) and two members for an average of 1 h per month (a cardiologist and a representative from the management) during the study.
  QI plan: The QI team can use CARDSS Online to grade indicators that the team aims to improve based on importance (five categories), feasibility (five categories), and expected time needed (three categories). CARDSS Online then ranks the indicators based on assigned ratings, and the team can select indicators for final inclusion in the QI plan. In principle, all quality indicators can be selected for improvement, but teams are encouraged to focus on a small subset of three to four indicators during each 3-month cycle. For each quality indicator included in the QI plan, users can specify the problem, presumed causes, improvement goal, and concrete actions on how to reach that goal. CARDDS Online does not provide tools, other than documentation, for systematic problem analysis or suggestions for improvement actions. For each action, the names of responsible team members and a deadline for achievement has to be entered.

**Table 2:** **Elements of the multifaceted guideline implementation intervention for both study arms per cardiac rehabilitation therapy**

**Study arm**	**CR therapy**	**Elements**	**Description of the elements**
A	Exercise training	CDS	Computerized decision support system at the point of care based on the most recent guidelines for CR
	Relaxation and stress management training	CDS	
	Education therapy	CDS	
		Feedback	Quarterly feedback reports on quality indicators for CR for arm A and B (see Table 3), monitoring of own performance over time, comprehensive benchmarking CR clinic’s performance to the other participating clinics
		Educational outreach visits	On site educational outreach visits after sending the feedback reports, supporting discussion of feedback results within a local QI team, supporting this team to define, implement and monitor a QI plan by means of a web-based QI system (CARDSS Online)
	Lifestyle change therapy (excluding physical activity)	CDS	
		Feedback	
		Educational outreach visits	
B	Exercise training and physical activity	CDS	
		Feedback	
	Relaxation and stress management training	CDS	
		Feedback	
		Educational outreach visits	
	Education therapy	CDS	
	Lifestyle change therapy	CDS

Four to six weeks after the first outreach visit, a telephone call will take place with the chair of the local QI team to discuss and support resolution of possible problems that occur during the implementation of the QI plan. Three months after the first round of feedback, a new feedback report is composed and becomes available in the system. During, but also in between, outreach visits, local QI teams can access CARDSS Online to provide status updates on actions listed in the current QI plan.

During the study period of 1 year, all clinics will receive four feedback reports, four outreach visits, and (at least) one telephone call. All visits will be carried out by the same investigator (MvEV) who has a health sciences background; she has been involved in the development of the CR quality indicator set [24] and has several years of experience with CR guideline implementation [41].

#### CARDSS online

The system was designed as a web-based application which can be consulted by all participating CR clinics. We designed the system to be primarily employed during educational outreach visits with the clinic’s QI teams. At the server side, CARDSS Online consists of a Microsoft SQL Server database and a Java web application. At the client side, clinics can use any web browser that is capable of rendering HTML and executing JavaScript. Development of the system and the architecture are described in more detail elsewhere [39].

### Participants

#### Inclusion criteria (clinic level)

All CR clinics that use an EPR for CR with CDS during the CR needs assessment procedure and that are willing to dispose their data for research and to set up a QI team are eligible to participate in the study. There are 91 CR clinics in the Netherlands, the majority affiliated with hospitals [42]. Twelve clinics are located in specialized rehabilitation centers [42], who have regional functions and can treat simple but especially more complex referred patients. Both types of clinic work according to the same guidelines and are eligible to participate in the study.

In the Netherlands, there are two commercial vendors of CDS systems for CR that can be used for data collection. Both systems have been validated for their advice being completely consistent with the guidelines. Also data collection in these systems is in line with the minimal dataset of our study. The minimal dataset is based on the set of 18 quality indicators for CR (see Table 3) and consists of (i) patient identification data (31 items), (ii) CR needs assessment data (80–130 items, see Table 4), (iii) data on selected rehabilitation goals and therapies (79 items), and (iv) CR evaluation data (105 items). Clinics that use one of the CDS systems automatically collect the right data items. The price of both systems depends on contract conditions, but amounts on average to €20,000 for purchase and to €4,000 per year for service, updates, and maintenance.

**Table 4:** **Items which need to be measured during the needs assessment procedure according to recommendations in the Cardiac Rehabilitation Guidelines** [26,38]

**Domain**	**Item**	**Clinical algorithm cardiac rehabilitation 2012**
1. Physical functioning	Objective exercise capacity	Maximal symptom limited exercise tolerance test.
		For patients with heart failure: completed with a spiroergometry (VO2max) test [43].
	Subjective exercise capacity	MacNew Quality-of-Life questionnaire (27 questions) [44,45]
2. Psychological functioning	Emotional function	MacNew Quality-of-Life questionnaire (27 questions) [44,45]
	Anxiety and Depression	Option 1: Generalized Anxiety Disorder scale (GAD-7, 7 questions) [46] in combination with the Patient Health Questionnaire (PHQ-9, 9 questions) [47]
		Option 2: Beck Anxiety Inventory (BAI, 21 questions) [48] in combination with the Beck Depression Inventory (BDI, 21 questions) [49]
		Option 3: Hospital Anxiety and Depression Scale (HADS, 14 questions) [50]
3. Social functioning	Social function	MacNew Quality-of-Life questionnaire (27 questions) [44,45]
	Social support	Option 1: Multidimensional Perceived Social Support Scale (MPSSS, 12 questions) [51,52]
		Option 2: ENRICHD Social Support Inventory (ESSI, 7 questions) [53]
	Life Partner	Clinical interview (3 questions)
	Resumption of work	Clinical interview (10 to 18 questions)
4. Cardiovascular risk profile	Cardiovascular risk profile	Physical examination (obesity, blood pressure), blood testing (cholesterol and diabetes)
5. Lifestyle factors	Smoking status	Clinical interview (1 to 4 questions) and specific treatment advice
	Physical activity	Monitor ‘Physical activity and Health’ (4 questions) [54]
	Dietary habits	Individual screening by dietician (in case of hypertension, hypercholesterolemia, obesity, or diabetes)
	Alcohol consumption	Five Shot questionnaire (5 questions) [55,56]

#### Inclusion criteria (patient level)

All consecutive CR patients that undergo the needs assessment procedure in one of the participating clinics will be included in the study. Clinics that participate in the study agree to enter all data of these patients in their EPR.

#### Recruitment

To promote participation and avoid volunteer effects, all CR clinics that use an EPR with CDS will receive a written invitation and, when they agree, will be visited by a member of the CARDSS team. During this introduction, the study protocol is explained to all team members, including the manager and cardiologist. Furthermore, the study is announced at national meetings of professional CR associations and during the 2-yearly national CR conference (visited by more than 350 professionals). After agreement on participation, the responsible administrator of the CR department (often the manager or the executive board of the hospital) must sign a consent form to formalize the organization’s commitment.

### Outcome measures

As in most guideline implementation studies, the proposed intervention is targeted at health care providers and is therefore expected to have a direct effect on process outcomes and to have only indirect, long-term effects on patient outcomes. The primary outcome measure will therefore be concordance, at patient level, to national CR guidelines with respect to the needs assessment (concordance of clinical decisions with the guidelines, for four group-based therapies). We will measure whether patients are treated according to the recommendations of the guidelines (i.e., treating patients who should have been treated and not treating patients who should have been untreated).

Secondary outcome measures are changes in performance of participating clinics as measured by 18 quality indicators for CR (see Table 3) and changes in practice variation on these indicators. Table 1 describes how the indicator set was developed [24].

### Data collection and validation

During the trial, participating CR clinics will use one of the two available commercial EPR systems with CDS. Each month, participating clinics are requested to extract data from their EPR, and submit it to the CARDSS Online system. After submission, the data are checked for validity and imported to the CARDSS Online database.

To measure data quality and completeness, a data audit will be conducted in each participating clinic at the end of the trial. To do so, we will ask access to an independent, local data source (preferably a digital agenda listing appointments for all therapies offered to CR patients). First, we will use this data source to check if our registration of therapies is correct, i.e., whether these therapies were indeed attended by the patient. To this end, 20 patients randomly selected from our database will be looked up in the independent data source. Second, we will use this source to check if our database is complete, i.e., whether all data of CR patients treated at the clinic also exist in our database (no missing patients). Therefore, 20 patients randomly selected from the independent source will be looked up in our database.

When no independent data source is available, we will ask the clinic in question to interview ten randomly selected patients over the telephone about the therapies they have followed and to check if their answers match our database. Thereafter, we will ask the clinics if we can interview ten randomly selected patients during a therapy session to see if their data is present in our database and if the therapy programs match.

If two or more of the selected patients from the independent source cannot be found in our database or if discrepancies in therapeutic data exist in more than five records, we will consider all the data of the clinic in question to be unreliable and exclude that clinic from the analyses.

### Sample size

To calculate the minimally required number of CR clinics participating in the trial, data from a previous trial (concerning the effect of CDS on guideline concordance) was used [7]. Calculations were based on the normal approximation to the binomial distribution, using a type I error risk (alpha) of 5% and 80% power. Also, we assumed that participating clinics will see 350 CR patients, on average, during the study period of 1 year, and that there will be a design effect of 23.0 due to clustering. This is based on an intra-cluster correlation coefficient (ICC) of 0.063, which is the median ICC for process variables found in 21 studies, reported by Campbell et al. [57].

In the previous study [7], CDS increased concordance with guideline-recommended therapeutic decisions for exercise training from 84.7% to 92.6%. To demonstrate a further increase to 97.6% (+5%) in the current study, at least 19 participating clinics (6,712 patients) are required under the assumptions given. For education, the concordance level previously rose from 63.9% to 87.6%. Demonstrating a further increase to 95.1% (+7.5%) requires 14 participating clinics (5,057 patients). For relaxation therapy, the previous study showed an increase from 34.1% to 59.6%, and showing an additional increase to 74.6% (+15%) requires ten participating clinics (3,517 patients). For lifestyle change therapy, finally, no significant effect of CDS was observed in the previous study (57.4% vs. 54.1%). To prove that the current intervention causes an increase from 57.4% to 72.4% (+15%) will also require that 13 clinics (3,632 patients) participate. Based on these results, we aim to include at least 19 CR clinics in our study.

### Randomization and allocation

We will randomly allocate CR clinics (clusters) to one of the both study arms, stratified by the number of patients per month entering the CR program: small (up to 30 patients) versus large (30 patients or more). Allocation is based on randomization with variable block size (two or four), performed with dedicated computer software written in the statistical programming language R (version 2.13.1). To conceal allocation, the software is used to generate a list of unique codes for both strata, where each code corresponds to an allocation (study arm A or B).

Three researchers will be involved in the allocation procedure. When a clinic is willing to participate, the first researcher (MvEV) will determine its stratum and communicate that to a second researcher (NdK) (without naming the clinic involved). The second researcher will look up the next unique code on the list for this stratum and pass that code to a third researcher who can determine the study arm, based on the given code. Using this procedure, both the second and third researchers are fully blinded for the allocation of clinics to the study arms during the procedure. The first researcher is not blinded but can however during this procedure not influence the allocation. Due to the characteristics of the intervention, it will not be possible to blind participants or the investigators providing the intervention.

### Statistical analysis

For the primary outcome measures, we will use mixed-effect logistic regression analysis [7,58], including clinic as random effect, to assess the effect of the intervention. Included covariates will be study arm, time since study start, and time × arm interaction. We will focus on the interaction term to assess the difference in change over the study period between the two arms—that is, the effect of the intervention—because we expect concordance to improve gradually.

For the secondary outcome measures, a similar analysis will be conducted, though replacing the logistic function by another link function as appropriate. Changes in practice variation will be assessed by including a random coefficient for ‘time since study start’ in the regression model and comparing the variation in estimated quality scores before and after the study.

### Process evaluation

The quantitative trial results will be completed by a qualitative process evaluation to assess experiences with the implementation of our intervention from participating CR clinics. To this end, we will employ a semi-structured qualitative research method known as concept mapping [59]. This method typically consists of the following five steps: In the first step (preparation), the research team decides on an open-ended focus question. In our study, this will likely be a question such as ‘Which circumstances facilitate systematic QI with CARDSS Online in your clinic?’ In the second step (generation), representatives of the QI teams of participating clinics meet in focus group sessions (six to eight persons) and are asked to develop a set of statements that address the focus. We note that the concept-mapping method does not require that consensus is sought during the focus group sessions; the participants may disagree and conflicting statements may come out.

In the third step (structuring), all QI team members of participating clinics are asked to structure the statements from the previous step by grouping them into categories that make sense to them and by rating them by importance and feasibility. In our study, this step is conducted over the internet with dedicated software (Concept Systems software version 4.0 [60]). During the fourth step (representation), an analysis algorithm implemented in the same software takes the collected grouping and rating data and generates a visual conceptual map using multidimensional scaling and hierarchical cluster analysis. In the fifth step (interpretation), labels and interpretations for the various parts of the map are developed.

### Ethics

The Institutional Review Board (IRB) of the Academic Medical Center (Amsterdam, the Netherlands) waived formal IRB approval. Our study database is registered according to the Dutch Personal Data Protection Art. In January 2012, the study was registered under the acronym ‘CARDSS-II trial’ in the Dutch Trial Register (NTR3251) [61].

## Discussion

### Study aim and hypothesis

The aim of this cluster randomized trial is to assess the effectiveness of a web-based QI system with indicator-based performance feedback and educational outreach visits to overcome organizational barriers for guideline concordance in multidisciplinary teams in the field of CR in the Netherlands. As the study will be conducted in CR clinics already using an EPR with CDS at the point of care, the intervention is compared to receiving this EPR with CDS alone. We hypothesize that our intervention will enable multidisciplinary CR teams to successfully target organizational barriers to improving guideline concordance. The results of this study are expected in 2015.

### Strengths and limitations

A unique feature of our study is that we provide performance feedback within a web-based system that incorporates all important QI concepts. The system supports local QI team teams to follow all steps of the QI process (monitoring indicator-based performance; selecting aspects of care which need improvement and developing a QI plan), resulting in explicit improvement goals with concrete actions and a time schedule. The outreach visits further increase active involvement in the process as the teams are encouraged to regularly meet and to specify goals they see as importantly attainable. To our knowledge, this is the first study to rigorously evaluate the effect of such an intervention in multidisciplinary teams. Second, we developed our multifaceted intervention based on an extensive analysis of barrier (see Table 1). During this analysis, persisting organizational barriers in the field of CR were revealed after the introduction of an EPR with CDS. We tailored and pilot-tested our intervention to specifically address these barriers during educational outreach visits with the multidisciplinary CR teams. During the visits, we specifically involved their managers in the discussion on the performance feedback and the implementation of QI actions. Third, we optimized agreement of CR professionals with the indicator set. This set was developed based on national guidelines and evidence from international literature and in close collaboration with representatives from all disciplines involved in CR [24] in the Netherlands. Fourth, as participating clinics are already working with the EPR with CDS functionality, they do not need to change their workflow for data collection and participation in the study. Finally, by using a balanced block-design with both study arms receiving part of the intervention, we minimize the risk that clinics lose their motivation to participate. We expect that this, in combination with the minimal workflow disruption, will maximize CR clinics’ willingness to participate and to minimize loss to follow-up.

Our study design also has some limitations. First, only CR clinics that use an EPR with CDS that facilitates registration of our minimal dataset are eligible to participate. Second, these clinics should be willing to dispose their data for research and to allocate resources to set up a QI team (volunteer bias). These two criteria may lead to the selection of a non-representative sample of CR clinics because eligible clinics are less likely to be understaffed and more likely to have information technology support to facilitate routine collection of CR data. The generalizability of our results will thus be limited to CR clinics that are motivated and equipped to systematically monitor and improve the quality of care they deliver. The essence of our intervention is that QI teams are free to formulate any improvement action, including those not specifically targeted at improving concordance to a specific guideline recommendation. Although this maximizes the involvement of the team in the improvement process and the commitment to goal attainment, it weakens the link between our intervention and primary outcome measure. By measuring changes in performance on the entire set of quality indicators as a secondary outcome, we aim to assess the direct relation between our intervention and performance changes. However, as is common in guideline implementation studies, these performance changes will only have indirect, long-term effects on patient outcomes. Fourth, the block randomization might cause underestimation of the effect size as clinics might start to improve both CR domains and not just the domain covered in their study arm because the intervention has raised their awareness for QI. Finally, the feedback on quality indicators will not automatically be corrected for differences in patient mix. However, during the trial, participating clinics can always request additional analyses on their data like, e.g., correction for patient mix to interpret their quality indicator results.

### Potential implications for practice and future research

Our study has both potential implications for practice and future research. First, it provides a better understanding of factors facilitating implementation of guidelines in multidisciplinary care teams. The results of our study may inform similar initiatives in other medical domains on how to use indicator-based performance feedback and outreach visits for improving the quality of care.

Second, it may influence the practice of CR and secondary prevention of cardiovascular disease in the Netherlands. The Dutch Health Care Inspectorate has recently demanded CR clinics to improve the quality of their programs based on the results of a quality assessment under all CR clinics in the Netherlands [42]. When our intervention appears to be successful, the Netherlands Society of Cardiology (NVVC) may decide to promote a national implementation of our intervention to meet the Inspectorate’s demand. In addition the process evaluation may result in valuable pointers to take into account when continuing the intervention. As such, the results of the study are relevant for all health care professionals and their organizations involved in cardiac aftercare in the Netherlands.

Finally, the results will guide future research that aims to identify success factors of feedback interventions. Our web-based QI system incorporates indicator-based performance feedback with involvement of multidisciplinary QI teams in developing and monitoring a local QI plan with explicit goals and is supported by educational outreach visits. Quantifying the effect of our intervention together with the qualitative data from the process evaluation will contribute to knowledge on potential barriers to using indicator-based feedback for improving the quality of care and how they can be overcome effectively.

## Appendix I

### Rules to set icon colors in CARDSS Online

- Structure (yes or no) indicators: The value ‘yes’ is considered acceptable (green), the value ‘no’ borderline (orange) if less than 50% of all clinics has yes, and poor (red) if more than 50% has yes.
- Frequency indicators (percentages): The interpretation is depending upon the mean value of all clinics. If this mean value is less than 66%, a value above 66% is considered acceptable (green); a value between 33% and 66% or a value up to 10% less than the value of all clinics is considered borderline (orange); and all other values are considered poor (red). If the mean value off all clinics is above 66%, a value up to 10% less than the value of all clinics is considered acceptable (green); a value above 33% is considered borderline (orange); and all other values are considered poor (red).
- Numeric non-frequency indicators (e.g., average increase in exercise capacity before and after the rehabilitation): Thresholds were determined by consulting the prevailing guidelines where possible, and otherwise based on observed distributions in previous studies or determined by consulting clinical experts. Also they depend on the mean value of all clinics. For instance, an average increase in exercise capacity above 10% is considered acceptable (green) if the mean value of all clinics is not above 20%; between 0% and 10% as borderline (orange) if the mean value of all clinics is not above 20%; and all other values, e.g., a decrease in exercise capacity, are considered poor (red).

## References

[1] Field MJ, Lohr KN (1990). Guidelines for Clinical Practice: Directions for a new Program.

[2] Grimshaw JM, Russell IT (1993). Effect of clinical guidelines on medical practice: a systematic review of rigorous evaluations. Lancet.

[3] Woolf SH, Grol R, Hutchinson A, Eccles M, Grimshaw J (1999). Clinical guidelines: potential benefits, limitations, and harms of clinical guidelines. BMJ.

[4] Grimshaw J, Eccles M, Tetroe J (2004). Implementing clinical guidelines: current evidence and future implications. J Contin Educ Health Prof.

[5] Goud R, Hasman A, Peek N (2008). Development of a guideline-based decision support system with explanation facilities for outpatient therapy. Comput Methods Programs Biomed.

[6] Goud R, Hasman A, Strijbis AM, Peek N (2009). A parallel guideline development and formalization strategy to improve the quality of clinical practice guidelines. Int J Med Inform.

[7] Goud R, de Keizer NF, ter RG, Wyatt JC, Hasman A, Hellemans IM, Peek N (2009). Effect of guideline based computerised decision support on decision making of multidisciplinary teams: cluster randomised trial in cardiac rehabilitation. BMJ.

[8] Goud R, van Engen-Verheul M, de Keizer NF, Bal R, Hasman A, Hellemans IM, Peek N (2010). The effect of computerized decision support on barriers to guideline implementation: a qualitative study in outpatient cardiac rehabilitation. Int J Med Inform.

[9] Cabana MD, Rand CS, Powe NR, Wu AW, Wilson MH, Abboud PA, Rubin HR (1999). Why don’t physicians follow clinical practice guidelines? A framework for improvement. JAMA.

[10] Grimshaw JM, Thomas RE, MacLennan G, Fraser C, Ramsay CR, Vale L, Whitty P, Eccles MP, Matowe L, Shirran L, Wensing M, Dijkstra R, Donaldson C (2004). Effectiveness and efficiency of guideline dissemination and implementation strategies. Health Technol Assess.

[11] Bright TJ, Wong A, Dhurjati R, Bristow E, Bastian L, Coeytaux RR, Samsa G, Hasselblad V, Williams JW, Musty MD, Wing L, Kendrick AS, Sanders GD, Lobach D (2012). Effect of clinical decision-support systems: a systematic review. Ann Intern Med.

[12] Roshanov PS, Misra S, Gerstein HC, Garg AX, Sebaldt RJ, Mackay JA, Weise-Kelly L, Navarro T, Wilczynski NL, Haynes RB, CCDSS Systematic Review Team (2011). Computerized clinical decision support systems for chronic disease management: a decision-maker-researcher partnership systematic review. Implement Sci.

[13] Roshanov PS, Fernandes N, Wilczynski JM, Hemens BJ, You JJ, Handler SM, Nieuwlaat R, Souza NM, Beyene J, Van Spall HG, Garg AX, Haynes RB (2013). Features of effective computerised clinical decision support systems: meta-regression of 162 randomised trials. BMJ.

[14] Tucker A, Edmondson A (2003). Why hospitals don’t learn from failures: organizational and psychological dynamics that inhibit system change. Calif Manage Rev.

[15] Davies HTO, Powell AE, Rushmer RK (2007). Healthcare Professionals’ Views on Clinician Engagement in Quality Improvement: a Literature Review.

[16] Langley GL, Nolan KM, Nolan TW, Norman CL, Provost LP (2009). The Improvement Guide: a Practical Approach to Enhancing Organizational Performance (2nd Edition).

[17] Ivers N, Jamtvedt G, Flottorp S, Young JM, Odgaard-Jensen J, French SD, O'Brien MA, Johansen M, Grimshaw J, Oxman AD (2012). Audit and feedback: effects on professional practice and healthcare outcomes. Cochrane Database Syst Rev.

[18] Wensing M, van der Weijden T, Grol R (1998). Implementing guidelines and innovations in general practice: which interventions are effective?. Br J Gen Pract.

[19] NHS Centre for Reviews and Dissemination (1999). Getting Evidence into Practice. Effective Health Care.

[20] Grol R, Grimshaw J (2003). From best evidence to best practice: effective implementation of change in patients’ care. Lancet.

[21] Francke AL, Smit MC, de Veer AJ, Mistiaen P (2008). Factors influencing the implementation of clinical guidelines for health care professionals: a systematic meta-review. BMC Med Inform Decis Mak.

[22] van Engen-Verheul M, de Keizer N, Hellemans I, Kraaijenhagen R, Hasman A, Peek N (2010). Design of a continuous multifaceted guideline-implementation strategy based on computerized decision support. Stud Health Technol Inform.

[23] van Engen-Verheul MM, Kemps HM, de Keizer NF, Hellemans IM, Goud R, Kraaijenhagen RA, Peek N (2012). Revision of the Dutch clinical algorithm for assessing patient needs in cardiac rehabilitation based on identified implementation problems. Eur J Cardiovasc Prev Rehabil.

[24] van Engen-Verheul M, Kemps H, Kraaijenhagen R, de Keizer N, Peek N (2011). Modified rand method to derive quality indicators: a case study in cardiac rehabilitation. Stud Health Technol Inform.

[25] Campbell BF, Sengupta S, Santos C, Lorig KR (1995). Balanced incomplete block design: description, case study, and implications for practice. Health Educ Q.

[26] Rehabilitation Committee (2011). Netherlands Society for Cardiology (NVVC) and Netherlands Heart Foundation (NHS) (both Guidelines on Cardiac Rehabilitation 2004) and Working Group PAAHR (partial revision 2011): Multidisciplinary Guidelines for Cardiac Rehabilitation (in Dutch).

[27] Centers for Disease Control and Prevention (CDC) (2008). Receipt of outpatient cardiac rehabilitation among heart attack survivors—United States, 2005. JAMA.

[28] Ades PA (2001). Cardiac rehabilitation and secondary prevention of coronary heart disease. N Engl J Med.

[29] Bethell H, Dalal H, Lewin R (2009). Cardiac rehabilitation in the United Kingdom. Heart.

[30] World Health Organization (1993). Needs and Action Priorities in Cardiac Rehabilitation and Secondary Prevention in Patients with CHD.

[31] Thomas RJ, King M, Lui K, Oldridge N, Pina IL, Spertus J, Bonow RO, Estes NA, Goff DC, Grady KL, Hiniker AR, Masoudi FA, Radford MJ, Rumsfeld JS, Whitman GR, AACVPR; ACC; AHA; American College of Chest Physicians; American College of Sports Medicine; American Physical Therapy Association; Canadian Association of Cardiac Rehabilitation; European Association for Cardiovascular Prevention and Rehabilitation; Inter-American Heart Foundation; National Association of Clinical Nurse Specialists; Preventive Cardiovascular Nurses Association; Society of Thoracic Surgeons (2007). AACVPR/ACC/AHA 2007 performance measures on cardiac rehabilitation for referral to and delivery of cardiac rehabilitation/secondary prevention services endorsed by the American College of Chest Physicians, American College of Sports Medicine, American Physical Therapy Association, Canadian Association of Cardiac Rehabilitation, European Association for Cardiovascular Prevention and Rehabilitation, Inter-American Heart Foundation, National Association of Clinical Nurse Specialists, Preventive Cardiovascular Nurses Association, and the Society of Thoracic Surgeons. J Am Coll Cardiol.

[32] Piepoli MF, Corra U, Benzer W, Bjarnason-Wehrens B, Dendale P, Gaita D, McGee H, Mendes M, Niebauer J, Zwisler AD, Schmid JP, Cardiac Rehabilitation Section of the European Association of Cardiovascular Prevention and Rehabilitation (2010). Secondary prevention through cardiac rehabilitation: from knowledge to implementation. A position paper from the Cardiac Rehabilitation Section of the European Association of Cardiovascular Prevention and Rehabilitation. Eur J Cardiovasc Prev Rehabil.

[33] Leon AS, Franklin BA, Costa F, Balady GJ, Berra KA, Stewart KJ, Thompson PD, Williams MA, Lauer MS, American Heart Association; Council on Clinical Cardiology (Subcommittee on Exercise, Cardiac Rehabilitation, and Prevention); Council on Nutrition, Physical Activity, and Metabolism (Subcommittee on Physical Activity); American association of Cardiovascular and Pulmonary Rehabilitation (2005). Cardiac rehabilitation and secondary prevention of coronary heart disease: an American Heart Association scientific statement from the Council on Clinical Cardiology (Subcommittee on Exercise, Cardiac Rehabilitation, and Prevention) and the Council on Nutrition, Physical Activity, and Metabolism (Subcommittee on Physical Activity), in collaboration with the American association of Cardiovascular and Pulmonary Rehabilitation. Circulation.

[34] Corra U, Piepoli MF, Carre F, Heuschmann P, Hoffmann U, Verschuren M, Halcox J, Giannuzzi P, Saner H, Wood D, Piepoli MF, Corrà U, Benzer W, Bjarnason-Wehrens B, Dendale P, Gaita D, McGee H, Mendes M, Niebauer J, Zwisler AD, Schmid JP, Document Reviewers (2010). Secondary prevention through cardiac rehabilitation: physical activity counselling and exercise training: key components of the position paper from the Cardiac Rehabilitation Section of the European Association of Cardiovascular Prevention and Rehabilitation. Eur Heart J.

[35] Muller-Riemenschneider F, Meinhard C, Damm K, Vauth C, Bockelbrink A, Greiner W, Willich SN (2010). Effectiveness of nonpharmacological secondary prevention of coronary heart disease. Eur J Cardiovasc Prev Rehabil.

[36] Bjarnason-Wehrens B, McGee H, Zwisler AD, Piepoli MF, Benzer W, Schmid JP, Dendale P, Pogosova NG, Zdrenghea D, Niebauer J, Mendes M, Cardiac Rehabilitation Section European Association of Cardiovascular Prevention and Rehabilitation (2010). Cardiac rehabilitation in Europe: results from the European Cardiac Rehabilitation Inventory Survey. Eur J Cardiovasc Prev Rehabil.

[37] van Engen-Verheul M, de Vries H, Kemps H, Kraaijenhagen R, de Keizer N, Peek N (2013). Cardiac rehabilitation uptake and its determinants in the Netherlands. Eur J Prev Cardiol.

[38] van Engen-Verheul MM, de Rijk A, Peek N (2012). Dutch Clinical Algorithm for Assessment of Patient Needs in Cardiac Rehabilitation and Secondary Prevention (in Dutch).

[39] van Engen-Verheul MM, van der Veer SN, de Keizer NF, Tjon Sjoe SW, van der Zwan EP, Peek N (2013). A Web-based system to facilitate local, systematic quality improvement by multidisciplinary care teams: development and first experiences of CARDSS online. Stud Health Technol Inform.

[40] Locke EA, Latham GP (2002). Building a practically useful theory of goal setting and task motivation. A 35-year odyssey. Am Psychol.

[41] van Engen-Verheul M, de Keizer N, Hellemans I, Kraaijenhagen R, Hasman A, Peek N (2010). Design of a continuous multifaceted guideline-implementation strategy based on computerized decision support. Stud Health Technol Inform.

[42] Reulings PG, van der Lans S (2012). Cardiac rehabilitation with lifestyle counselling after myocardial infarction: it helps, but not everyone undergoes it. Ned Tijdschr Geneeskd.

[43] Mezzani A, Agostoni P, Cohen-Solal A, Corra U, Jegier A, Kouidi E, Mazic S, Meurin P, Piepoli M, Simon A, Laethem CV, Vanhees L (2009). Standards for the use of cardiopulmonary exercise testing for the functional evaluation of cardiac patients: a report from the Exercise Physiology Section of the European Association for Cardiovascular Prevention and Rehabilitation. Eur J Cardiovasc Prev Rehabil.

[44] Dixon T, Lim LL, Oldridge NB (2002). The MacNew heart disease health-related quality of life instrument: reference data for users. Qual Life Res.

[45] de Gucht V, van Elderen T, van der Kamp L, Oldridge N (2004). Quality of life after myocardial infarction: translation and validation of the MacNew Questionnaire for a Dutch population. Qual Life Res.

[46] Spitzer RL, Kroenke K, Williams JB, Lowe B (2006). A brief measure for assessing generalized anxiety disorder: the GAD-7. Arch Intern Med.

[47] Spitzer RL, Kroenke K, Williams JB (1999). Validation and utility of a self-report version of PRIME-MD: the PHQ primary care study. Primary Care Evaluation of Mental Disorders. Patient Health Questionnaire. JAMA.

[48] Beck AT, Epstein N, Brown G, Steer RA (1988). An inventory for measuring clinical anxiety: psychometric properties. J Consult Clin Psychol.

[49] Beck AT, Steer RA, Ball R, Ranieri W (1996). Comparison of beck depression inventories -IA and -II in psychiatric outpatients. J Pers Assess.

[50] Spinhoven P, Ormel J, Sloekers PP, Kempen GI, Speckens AE, Van Hemert AM (1997). A validation study of the Hospital Anxiety and Depression Scale (HADS) in different groups of Dutch subjects. Psychol Med.

[51] Zimet GD, Powell SS, Farley GK, Werkman S, Berkoff KA (1990). Psychometric characteristics of the Multidimensional Scale of Perceived Social Support. J Pers Assess.

[52] Pedersen SS, Spinder H, Erdman RA, Denollet J (2009). Poor perceived social support in implantable cardioverter defibrillator (ICD) patients and their partners: cross-validation of the multidimensional scale of perceived social support. Psychosomatics.

[53] Mitchell PH, Powell L, Blumenthal J, Norten J, Ironson G, Pitula CR, Froelicher ES, Czajkowski S, Youngblood M, Huber M, Berkman LF (2003). A short social support measure for patients recovering from myocardial infarction: the ENRICHD Social Support Inventory. J Cardiopulm Rehabil.

[54] Douwes M, Hildebrandt VH (2000). Questions on the amount of physical activity. Geneeskunde en Sport.

[55] De Rick A, Vanheule S (2007). Pilot study: does the Five Shot Questionnaire give an indication of the severity of alcohol use-related problems?. Subst Use Misuse.

[56] Seppa K, Lepisto J, Sillanaukee P (1998). Five-shot questionnaire on heavy drinking. Alcohol Clin Exp Res.

[57] Campbell MK, Fayers PM, Grimshaw JM (2005). Determinants of the intracluster correlation coefficient in cluster randomized trials: the case of implementation research. Clin Trials.

[58] Pinheiro JC, Bates DM (2000). Mixed Effects Models in S and S-plus.

[59] Kane M, Trochim WMK (2007). Concept Mapping for Planning and Evaluation.

[60] Concept Systems Inc: *Concept Systems Software v 4.0*; 2013. [http://www.conceptsystems.com/content/view/the-concept-system.html], Date accessed 04-09-2013.

[61] Dutch Trial Register: *Implementation of the Cardiac Rehabilitation (CR) Guidelines by a Multifaceted Decision Support, Feedback and Outreach Visit Intervention: A Cluster Randomized Trial*; 2012. http://www.trialregister.nl/trialreg/admin/rctview.asp?TC=3251 (last accessed 01-06-2013).

